# Magnetic Resonance Imaging-Based Radiomics Analysis of Cervical Lymph Nodes Associated with Medication-Related Osteonecrosis of the Jaw

**DOI:** 10.3390/jimaging12100507

**Published:** 2026-10-13

**Authors:** Hirotaka Muraoka, Hiroshi Yamamoto, Chikara Awazu, Kotaro Ito

**Affiliations:** 1 Department of Radiology, Nihon University School of Dentistry at Matsudo, 2-870-1 Sakaecho-Nishi, Matsudo 271-8587, Chiba, Japan; 2 Department of Oral Surgery, Nihon University School of Dentistry at Matsudo, 2-870-1 Sakaecho-Nishi, Matsudo 271-8587, Chiba, Japan

**Keywords:** medication-related osteonecrosis of the jaw, magnetic resonance imaging, radiomics analysis

## Abstract

To quantitatively evaluate the magnetic resonance imaging (MRI) texture features of cervical lymph nodes (LNs) associated with medication-related osteonecrosis of the jaw (MRONJ). This retrospective exploratory study included 36 women (72 cervical LNs) with or without MRONJ who underwent MRI at our institution between April 2017 and April 2022. The participants were 58–90 years old (mean age, 77.7 years). Radiomic analysis was conducted using manually delineated regions of interest on short tau inversion recovery (STIR) images, from which 279 quantitative features were extracted with MaZda version 3.3. Data normality was evaluated using the Shapiro–Wilk test. Variables with a normal distribution were compared using the independent-samples *t*-test, whereas non-normally distributed variables were analyzed using the Mann–Whitney U test. *p* values were adjusted for multiple comparisons using the Benjamini–Hochberg false discovery rate (FDR) procedure. Receiver operating characteristic (ROC) curve analysis was performed to evaluate the discriminatory performance of the selected texture features. Twenty texture features were selected from the initial 279 features. All 10 selected texture features of the submandibular LNs yielded FDR-adjusted *p* values < 0.05, whereas none of the selected features of the superior internal jugular LNs met this threshold. ROC analysis demonstrated AUCs ranging from 0.67 to 0.82. MRI radiomics analysis may provide complementary quantitative information for evaluating lymph node alterations associated with MRONJ.

## 1. Introduction

Medication-related osteonecrosis of the jaw (MRONJ) is a serious complication associated with antiresorptive and antiangiogenic medications [1]. These are routinely used to reduce the risk of fractures in patients with osteoporosis and to inhibit metastasis in malignancy [2,3]. According to the diagnostic criteria proposed by the American Association of Oral and Maxillofacial Surgeons (AAOMS), MRONJ is diagnosed in patients who have received current or previous treatment with antiresorptive or antiangiogenic drugs, present with exposed bone or bone that can be probed through an intraoral or extraoral fistula in the maxillofacial region for more than 8 weeks, and have no history of radiation therapy to the jaws or jaw metastasis [4]. Clinical findings include purulent drainage and fistulas, redness and swelling of the surrounding tissues, and swollen lymph nodes [5]. Moreover, extensive osteonecrosis involving surrounding nerves can cause severe pain, mastication and swallowing disorders, and even pathological fractures. Hence, accurate evaluation including surrounding tissues is required.

Owing to its excellent soft-tissue contrast, magnetic resonance imaging (MRI) plays an important role in the evaluation of inflammatory changes involving the maxillofacial region. Furthermore, texture analysis, which has been attracting attention in recent years, can detect subtle changes in gray-level patterns within specific regions. MRI texture analysis enables the extraction and quantification of features such as histogram-based parameters, skewness, kurtosis, entropy, and gray-level co-occurrence matrix (GLCM) features. Many studies have employed this technique [6,7,8]. Recent studies have investigated the application of radiomics to MRONJ using panoramic radiography and computed tomography (CT) [9,10]. These studies have demonstrated the potential of radiomic features for predicting MRONJ development. Previous studies have also explored radiomics for the characterization of inflammatory and reactive cervical lymph nodes [11]. In particular, MRI-derived radiomics has been investigated for differentiating reactive hyperplasia, suppurative lymphadenitis, Kikuchi disease, and malignant lymphadenopathy [12]. However, previous MRONJ radiomics studies have primarily focused on the jawbone, and the radiomic characteristics of regional cervical lymph nodes associated with MRONJ remain unclear.

Therefore, this study aimed to investigate the quantitative MRI radiomic characteristics of cervical lymph nodes in patients with MRONJ. We hypothesized that MRI-derived radiomic features would differ between MRONJ-associated and control lymph nodes.

## 2. Methods

### 2.1. Patients

This retrospective study was approved by the Ethics Committee of Nihon University School of Dentistry at Matsudo (Approval No. EC21-003; approved on 10 May 2021). Informed consent for participation was waived by the Ethics Committee because of the retrospective nature of the study. All procedures were conducted in accordance with the ethical principles of the Declaration of Helsinki (1975, revised in 2013) [13].

Patients diagnosed with MRONJ who reported symptoms of pain in the mandible and underwent MRI at our hospital between April 2017 and April 2022 were eligible for inclusion. Patients with severe susceptibility artifacts or a history of radiotherapy were excluded. The diagnosis of MRONJ was based on the AAOMS position paper [4]. The details of the MRONJ stage definition are as follows:

(1) **Stage 0**—Patients with no clinical evidence of necrotic bone but with nonspecific symptoms or clinical and radiographic findings suggestive of MRONJ.
(2) **Stage 1**—Patients with exposed necrotic bone or a fistula communicating with bone, but without symptoms or clinical signs of infection.
(3) **Stage 2**—Patients with exposed necrotic bone or a bone-probing fistula accompanied by infection, characterized by pain and erythema around the exposed bone, with or without purulent discharge.
(4) **Stage 3**—Patients with exposed necrotic bone or a fistula extending to bone, accompanied by pain and infection, together with one or more of the following findings: necrotic bone extending beyond the alveolar process (e.g., to the mandibular inferior border or ramus, or to the maxillary sinus or zygoma), pathological fracture, extraoral fistula, oroantral or oronasal communication, or osteolysis reaching the mandibular inferior border or the floor of the maxillary sinus.

The control group consisted of age- and sex-matched outpatients who underwent MRI for evaluation of temporomandibular disorders and showed no evidence of inflammatory disease in the jaw. None of the control participants had a history of antiresorptive or antiangiogenic medication use. LNs in the cervical region fell into two groups: submandibular LNs and superior internal jugular LNs. The classification of these LNs was based on the Rouviere and the Som et al. level system [14,15,16].

### 2.2. Data Collection

MRI examinations were performed using a 1.5-T superconducting scanner (Intera Achieva^®^ 1.5 T Nova, Philips Medical Systems, Best, The Netherlands) equipped with a five-channel phased-array coil. Short tau inversion recovery (STIR) images were obtained with a spin-echo sequence using the following imaging parameters: repetition time (TR), 2500 ms; echo time (TE), 60 ms; inversion time (TI), 180 ms; number of excitations (NEX), 2; and echo train length (ETL), 15. Additional acquisition parameters included a slice thickness of 6.0 mm, an interslice gap of 1.2 mm, a matrix size of 320 × 256, a field of view of 230 × 195.5 mm, and a single acquisition.

**Figure 1:**
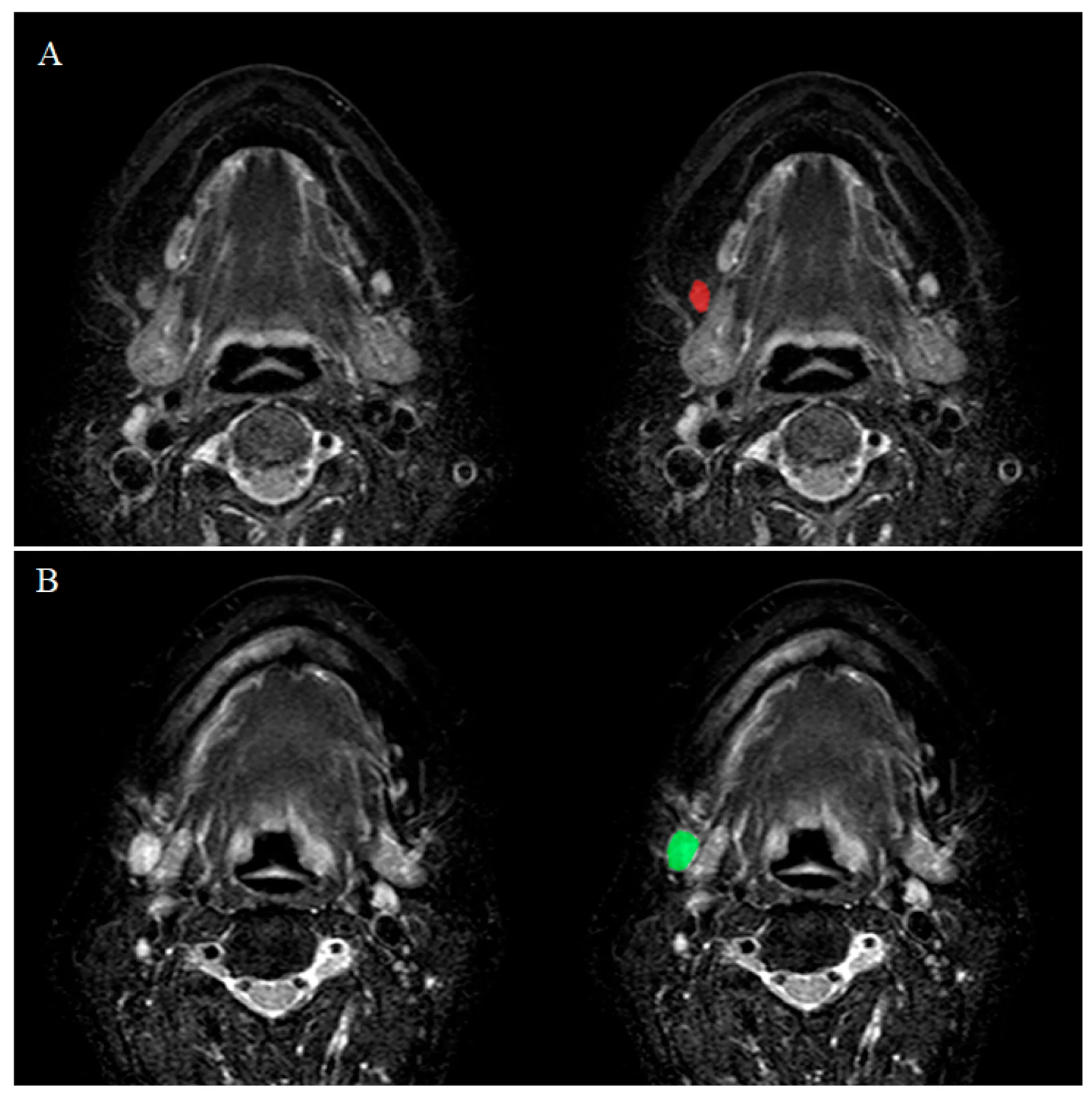
Regions of interest (ROI) were manually drawn on the short tau inversion recovery (STIR) magnetic resonance (MR) images of each LN with the largest short axis diameter ((**A**): Control group, (**B**): MRONJ group).

Quantitative image analysis was performed on STIR images using MaZda version 3.3, a software package developed for the extraction and evaluation of image texture features [17,18]. For each participant, one submandibular LN and one superior internal jugular LN were selected for analysis. In the MRONJ group, the LN with the largest short-axis diameter on the affected side was selected from each LN group, whereas, in the control group, the LN with the largest short-axis diameter from either side was selected from each LN group (Figure 1). The Regions of interests (ROIs) used for texture analysis were manually delineated by an oral radiologist with 11 years of experience. For assessment of interobserver reliability, the ROIs were independently delineated by a second oral radiologist. The ROI was placed within the visible nodal margin, with care taken to exclude adjacent adipose tissue, blood vessels, and other surrounding structures that could influence the measured signal-intensity distribution. The same ROI delineation and image-processing procedures were applied to all participants. Before radiomic feature extraction, the default intensity-range setting in MaZda was used, corresponding to the original image intensity range from 1 to (N_g_ = 2^k^), where (^k^) denotes the number of bits per pixel used for image encoding. No ±3 sigma or 1–99% intensity normalization was applied. For autoregressive model features, ROI-based standardization was performed automatically by MaZda, independently of the selected normalization setting. A total of 279 quantitative texture features were subsequently calculated from each ROI. Feature extraction included histogram-based, autoregressive model, gray-level run-length matrix (GLRLM), gray-level co-occurrence matrix (GLCM), absolute-gradient, and wavelet-derived parameters. GLRLM features were calculated using 6 bits per pixel in four directions (0°, 45°, 90°, and 135°). These features describe the lengths and distributions of consecutive pixels with the same gray-level value along a specified direction. GLCM features were also computed using 6 bits per pixel in four directions, with interpixel distances of 1, 2, 3, 4, and 5. GLCM-derived parameters characterize the spatial relationships between pairs of gray-level values within the ROI. Absolute-gradient features, which reflect local changes in signal intensity, were obtained using 4 bits per pixel, whereas wavelet features, which characterize image patterns across different spatial-frequency components, were calculated using 8 bits per pixel. Feature ranking was performed separately for the submandibular and superior internal jugular lymph-node groups using the feature-selection functions incorporated into MaZda. The ranking procedure was based on two criteria: Fisher coefficient and the probability of classification error combined with the average correlation coefficient (POE + ACC). A higher Fisher coefficient indicates greater separation between the MRONJ and control groups relative to the variability within each group. By contrast, a lower POE + ACC value identifies features associated with a lower estimated classification error and less redundancy with other features. For each lymph-node group, the top 10 features according to each ranking criterion were predefined for selection. The 10 highest-ranked features according to the Fisher coefficient and the 10 highest-ranked features according to POE + ACC were identified. Both ranking criteria selected the same 10 features, which were subsequently used for between-group comparisons and ROC analysis. The mathematical procedures underlying feature calculation and ranking have been described in the original reports on the MaZda software [17,18].

### 2.3. Statistical Analysis

Interobserver reliability of the selected texture feature values extracted from independently delineated ROIs was assessed using the intraclass correlation coefficient based on a two-way random-effects model (ICC [2,1]). Agreement was classified as poor (<0.50), moderate (0.50–0.75), good (0.75–0.90), or excellent (>0.90). The normality of the variables was assessed using the Shapiro–Wilk test. The homogeneity of variances was assessed using Levene’s test. Variables with a normal distribution were compared using Student’s *t*-test assuming equal variances, whereas non-normally distributed variables were analyzed using the Mann–Whitney U test. Group status (MRONJ vs. control) was treated as the explanatory variable, and the MRI texture features of the LNs were treated as the outcome variables. A ROC curve analysis was performed to exploratively assess the discriminatory performance of the selected MRI texture features, and the area under the curve (AUC) was calculated. Because feature selection and ROC analysis were performed using the same dataset, the AUCs were considered apparent (in-sample) estimates of discriminatory performance. The optimal cut-off point was determined by maximizing Youden’s index. These analyses were performed using a statistical package (version 28; IBM Japan Inc., Tokyo, Japan) and R (R 4.0.3, R Foundation for Statistical Computing, Vienna, Austria). The Benjamini–Hochberg false discovery rate (FDR) procedure was applied separately to the 10 selected features within each LN group. An FDR-adjusted *p* value < 0.05 was used as the threshold for identifying potentially significant differences in this exploratory analysis.

## 3. Results

This study initially identified 21 patients who were diagnosed with MRONJ and underwent MRI at our hospital between April 2017 and April 2022. Three patients were excluded because of severe susceptibility artifacts (*n* = 2) or a history of radiotherapy (*n* = 1). Consequently, 18 female patients with MRONJ (aged 58–90 years; mean age, 77.67 years) were included in the final analysis. The control group consisted of 18 age- and sex-matched female outpatients (aged 58–90 years; mean age, 77.67 years) who underwent MRI for evaluation of temporomandibular disorders and showed no evidence of inflammatory disease in the jaw. Among the 18 patients with MRONJ, 5, 5, 6, and 2 patients were classified as Stage 0, Stage 1, Stage 2, and Stage 3, respectively.

Table 1 provides a description of all extracted texture features. Texture features such as histogram, GLCM, GLRLM and wavelet transform were extracted.

Table 2 presents the exploratory comparisons of the 10 selected texture features of submandibular LNs between the control and MRONJ groups. All 10 features yielded FDR-adjusted *p* values below 0.05. Table 3 presents the corresponding results for superior internal jugular LNs, in which none of the 10 selected features had an FDR-adjusted *p* value below 0.05.

**Figure 2:**
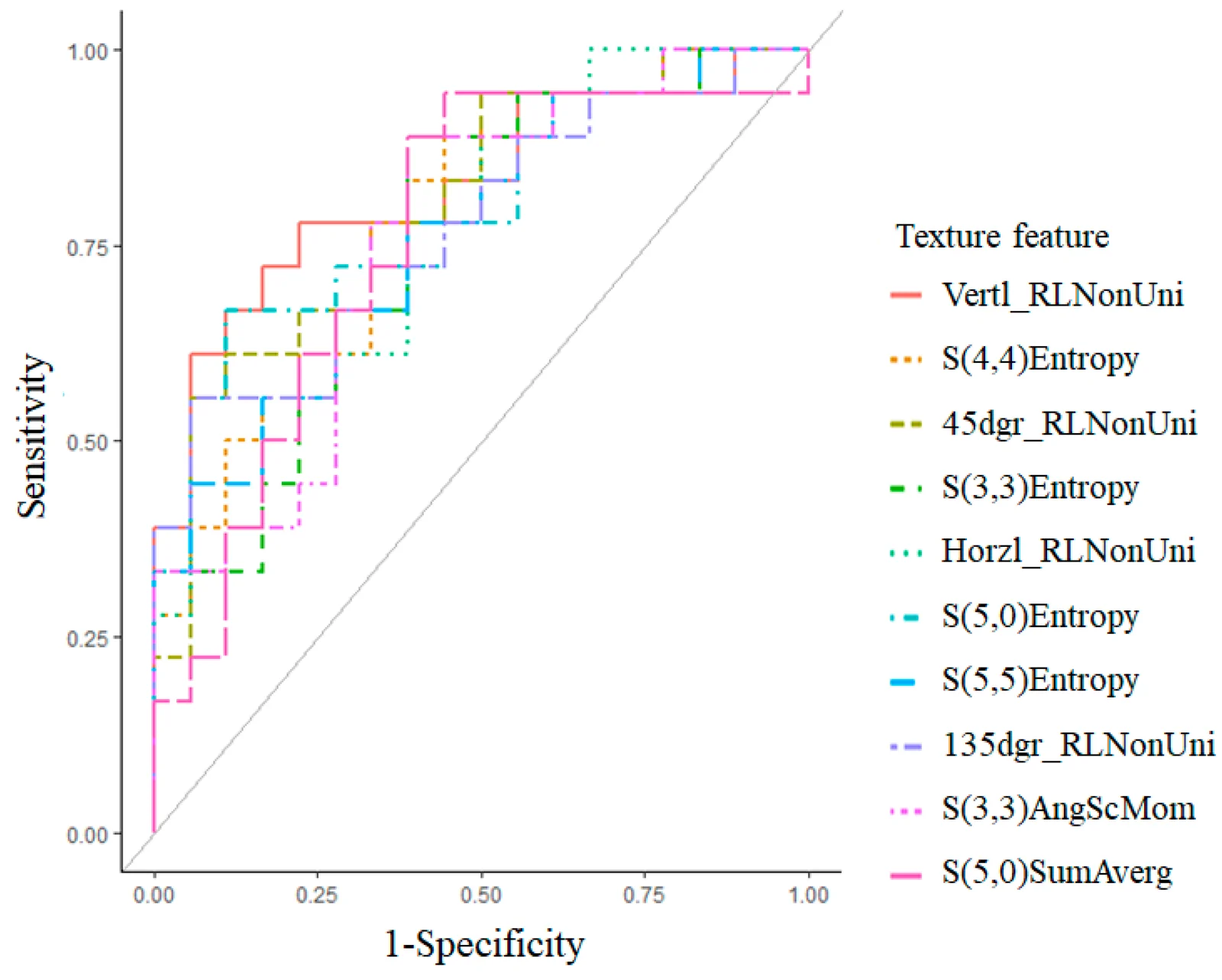
The graph depicts the ROC curves of the 10 selected texture features for distinguishing MRONJ-associated submandibular LNs from control LNs. The corresponding AUCs ranged from 0.76 to 0.82.

**Figure 3:**
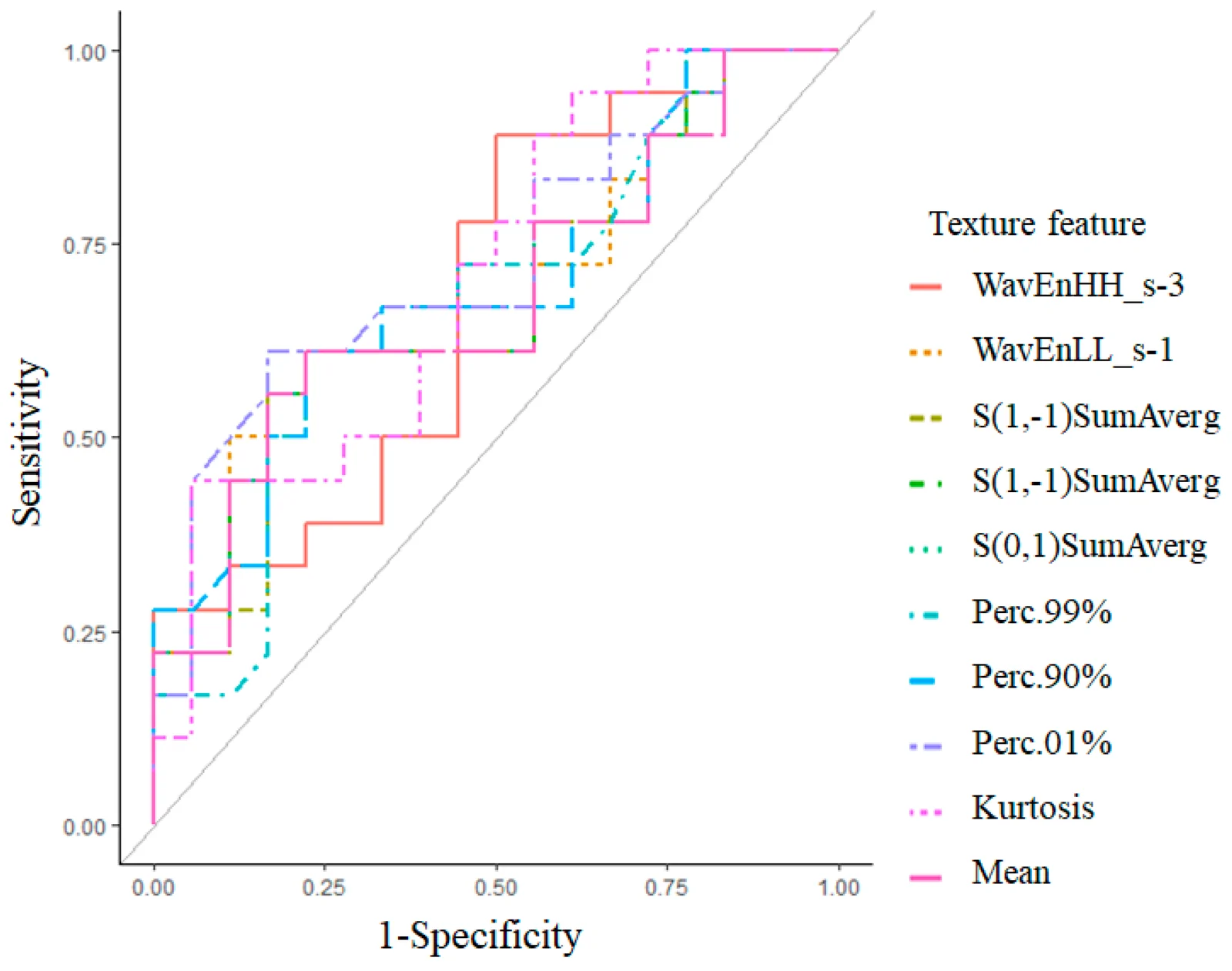
The graph depicts the ROC curves of the 10 selected texture features for distinguishing MRONJ-associated superior internal jugular LNs from control LNs. The corresponding AUCs ranged from 0.67 to 0.73.

ROC analysis revealed an AUC ranging from 0.76 to 0.82 for all texture features for distinguishing between control and MRONJ in submandibular LNs (Figure 2). In superior internal jugular LNs, the AUCs for all texture features ranged from 0.67 to 0.73 for distinguishing between control and MRONJ (Figure 3). The AUCs with 95% confidence intervals, optimal cut-off values determined using the Youden index, and corresponding sensitivity and specificity are presented in Table 4.

Interobserver reliability was excellent, with ICC values for the selected radiomic features ranging from 0.91 to 0.93 for the submandibular LNs and from 0.94 to 0.95 for the superior internal jugular LNs.

## 4. Discussion

This study identified differences in selected MRI texture features between MRONJ-associated and control submandibular LNs after FDR correction, whereas no corresponding differences were identified in superior internal jugular LNs.

Antiresorptive and antiangiogenic agents are widely prescribed for the prevention of skeletal-related events in patients with malignant disease and for the treatment of osteoporosis and other metabolic bone disorders. As the use of these medications continues to increase worldwide, the incidence of MRONJ has also risen accordingly [19,20]. Advanced MRONJ is frequently accompanied by persistent inflammation, secondary infection, and extensive soft-tissue involvement, resulting in pain, impaired mastication and swallowing, and a marked reduction in patients’ quality of life [21,22]. Because inflammatory changes often extend beyond the jawbone, comprehensive imaging assessment of both the surrounding soft tissues and regional cervical lymph nodes is clinically important. Although panoramic radiography and computed tomography are useful for evaluating osseous destruction, MRI provides superior soft-tissue contrast and is particularly sensitive for detecting bone marrow abnormalities and inflammatory changes [23,24,25,26]. However, to the best of our knowledge, no study has investigated the LNs affected by MRONJ using MRI image-based texture analysis. Texture analysis is a technique for extracting features that are unrecognizable to the human eye from specific areas of an image. A set of mathematical formulas is used to generate quantitative parameters that characterize the spatial variation in gray levels within the selected area of an image. This technique has been used in combination with various modalities [26,27,28]. Hence, we hypothesized that this imaging technique could provide quantitative information for assessing cervical LNs associated with MRONJ.

In the present study, several texture features differed between MRONJ-associated and control LNs. After FDR correction, differences were observed in the selected features of submandibular LNs, but not in those of superior internal jugular LNs. These findings suggest that MRI radiomics may provide quantitative information on signal-intensity distribution and tissue heterogeneity within regional LNs.

A histogram (Mean, Kurtosis, Percentile) indicates the intensity distribution of pixels on an image without considering spatial relationships. We found that MRONJ-associated LNs had a high proportion of high pixel intensities. Because STIR imaging is sensitive to changes in tissue water content, these findings suggest differences in the internal signal-intensity distribution of MRONJ-associated LNs.

GLCM (Entropy, Sum Average, Angular second moment) provides information about the distribution and spatial relationships of gray-level values between pairs of pixels separated by a defined distance in a given direction. Entropy and Angular second moment represent the randomness and uniformity of pixel intensities, respectively. These texture features differed between MRONJ-associated and control LNs, suggesting greater heterogeneity and reduced structural uniformity within MRONJ-associated LNs.

The Sum Average represents the mean of the gray-level sum distribution. The Sum Average of MRONJ-associated LNs was higher than that of control LNs, suggesting a greater contribution of relatively high signal intensities throughout the LN. These findings suggest that the observed signal-intensity alterations were distributed throughout the LN rather than being limited to localized areas.

GLRLM (Run-length nonuniformity) provides information about runs of pixels with the same gray-level values in a given direction and reflects intensity homogeneity. Run-length nonuniformity was higher in MRONJ-associated LNs than in control LNs, indicating greater variability in homogeneous gray-level runs and reduced structural uniformity. Such changes may reflect alterations in the internal architecture and signal heterogeneity of reactive LNs. However, the biological basis of these radiomic changes remains uncertain because histopathological correlation was not available in the present study. Wavelet transform also showed pixel intensity non-uniformity in MRONJ-associated LNs, suggesting that image heterogeneity may be present across different spatial-frequency components. Collectively, these findings suggest that MRONJ-associated alterations in regional LNs may be quantitatively characterized using multiple types of MRI texture features.

ROC analysis revealed AUC values ranging from 0.67 to 0.82. Although these values are insufficient for use as standalone diagnostic markers, they suggest that MRI texture features may provide complementary quantitative information regarding MRONJ-associated changes in cervical LNs. However, because no direct comparison with conventional visual MRI assessment was performed, the incremental value of radiomics over routine MRI evaluation remains unclear. Because feature selection and ROC analysis were performed using the same dataset, the reported AUCs represent apparent (in-sample) discriminatory performance and may be subject to feature-selection bias. Accordingly, these AUC values should not be interpreted as estimates of generalizable diagnostic performance. Therefore, given the exploratory nature of the present study and the lack of independent validation, further studies are required to confirm these findings and establish their potential clinical utility.

A limitation of our study was that, due to its retrospective design, only MR images acquired with a 6-mm slice thickness were available for analysis. This relatively thick slice thickness may have resulted in partial-volume effects, particularly in smaller lymph nodes, and may have influenced the extracted radiomic features. Furthermore, only one representative lymph node with the largest short-axis diameter from each lymph-node group was analyzed in each participant; therefore, this selection approach may have introduced sampling bias, and the findings may not reflect inflammatory changes in all regional cervical lymph nodes. Moreover, the sample size was relatively small, particularly in relation to the large number of radiomic features extracted, which may have increased the risk of feature-selection bias and optimism in the apparent discriminatory performance. In addition, all participants were female, limiting the generalizability of the findings to male patients. The retrospective, single-center design may limit the reproducibility and generalizability of the findings. Furthermore, patients with different MRONJ stages (Stages 0–3) were included, and stage-stratified analyses were not performed because of the limited number of patients in each stage. Therefore, we cannot exclude the possibility that disease severity and the extent of local inflammation or infection influenced the observed lymph-node radiomic features. Because the control group had no history of antiresorptive or antiangiogenic medication use, the observed differences may also reflect medication exposure or underlying diseases rather than MRONJ itself. Although FDR correction was applied to the selected features to account for multiple comparisons, the resulting FDR-adjusted *p* values should be interpreted as exploratory because the FDR correction does not account for potential bias introduced by data-driven feature selection. In addition, only individual radiomic features were evaluated, and no multivariable combined radiomic signature was developed. Further studies involving larger, independent cohorts including both male and female participants are required to confirm these findings.

In conclusion, MRI radiomics may provide complementary quantitative information for assessing cervical LN changes associated with MRONJ.

## Figures and Tables

**Table 1:** Description of texture features extracted in the analysis.

Feature Category	Description	Details of Features
Histogram	Describes the distribution of pixel intensities without considering spatial relationships.	Mean Kurtosis Perc.
GLCM	Describes the spatial distribution of pairs of gray-level values separated by a defined distance in a given direction.	Entropy SumAverg AngScMom
GLRLM	Describes runs of pixels with the same gray-level value in a given direction and reflects directional intensity homogeneity.	RLNonUni
Wavelet transform	Describes image characteristics in different spatial-frequency components using wavelet decomposition.	WavEnHH

Abbreviations: Perc.: percentile, GLCM: gray-level co-occurrence matrix, SumAverg: sum average, AngScMom: angular second moment; GLRLM: gray-level run-length matrix, RLNonUni: run-length nonuniformity, WavEnHH: wavelet energy HH.

**Table 2:** Comparison of texture features of submandibular lymph nodes between the control and MRONJ groups.

Texture Features	Control (*n* = 18)	MRONJ (*n* = 18)	*p*-Value	FDR-Adjusted *p*-Value *
GLCM				
S (3,3) Entropy	2.04 ± 0.2	2.25 ± 0.18	0.003	0.006
S (4,4) Entropy	2.0 ± 0.22	2.23 ± 0.2	0.002	0.006
S (5,0) Entropy	1.96 ± 0.26	2.21 ± 0.21	0.005	0.006
S (5,5) Entropy	1.91 ± 0.3	2.18 ± 0.23	0.004	0.006
S (3,3) AngScMom	0.012 [0.007–0.014]	0.0069 [0.005–0.0087]	0.006	0.006
S (5,0) SumAverg	58.47 ± 15.66	73.58 ± 14.53	0.005	0.006
GLRLM				
45dgr_RLNonUni	118.74 [87.69–159.29]	192.04 [146.52–224.51]	0.002	0.006
Vertl_RLNonUni	110.18 [79.17–134.37]	166.59 [141.28–213.0]	<0.001	0.006
135dgr_RLNonUni	123.27 [97.48–164.07]	201.5 [143.35–266.06]	0.005	0.006
Horzl_RLNonUni	115.22 [82.11–147.01]	171.5 [129.94–234.58]	0.004	0.006

SD: standard deviation; IQR: interquartile range; MRONJ: medication-related osteonecrosis of the jaw; GLCM: gray-level co-occurrence matrix; AngScMom: angular second moment; SumAverg: sum average; GLRLM: gray-level run-length matrix, RLNonUni: run-length nonuniformity. Data are presented as mean ± standard deviation (SD) or median [interquartile range (IQR)], as appropriate. * *p* values were adjusted for multiple comparisons using the Benjamini–Hochberg false discovery rate (FDR) procedure.

**Table 3:** Comparison of texture features of superior internal jugular nodes between the control and MRONJ groups.

Texture Features	Control (*n* = 18)	MRONJ (*n* = 18)	*p* Value	FDR-Adjusted *p* Value *
Histogram				
Perc.99%	182.5 [166.5–206.75]	218 [173–230]	0.064	0.064
Perc.90%	170.67 ± 29.77	192.5 ± 29.23	0.033	0.060
Perc.01%	73.67 ± 26.6	93.78 ± 26.62	0.030	0.060
Mean	142.24 ± 27.48	160.85 ± 25.86	0.044	0.060
Kurtosis	0.58 [0.05–1.39]	0.11 [−0.53–0.58]	0.031	0.060
GLCM				
S (1,−1) SumAverg	73.13 ± 14.12	82.51 ± 13.25	0.048	0.060
S (1,1) SumAverg	73.12 ± 14.07	82.49 ± 13.0	0.046	0.060
S (0,1) SumAverg	72.66 ± 13.89	82.06 ± 13.11	0.044	0.060
Wavelet transform				
WavEnHH_s-3	58.19 [38.91–102.61]	94.76 [67.48–161.6]	0.064	0.064
WavEnLL_s-1	19,881.41 ± 7176.83	24,983.58 ± 7563.73	0.046	0.060

SD: standard deviation; IQR: interquartile range; MRONJ: medication-related osteonecrosis of the jaw; Perc.: percentile; SumAverg: sum average; GLCM: gray-level co-occurrence matrix; WavEnHH: wavelet energy HH; WavEnLL: wavelet energy LL. Data are presented as mean ± standard deviation (SD) or median [interquartile range (IQR)], as appropriate. * *p* values were adjusted for multiple comparisons using the Benjamini–Hochberg false discovery rate (FDR) procedure.

**Table 4:** ROC analysis of selected texture features for distinguishing MRONJ-associated lymph nodes from control lymph nodes.

Texture Feature	AUC (95% CI)	Cut-Off	Sensitivity (%)	Specificity (%)
Submandibular LNs				
Vertl_RLNonUni	0.824 (0.685–0.963)	151.49	66.7	88.9
S (4,4) Entropy	0.787 (0.640–0.934)	2.10	77.8	66.7
45dgr_RLNonUni	0.799 (0.655–0.944)	171.94	61.1	88.9
S (3,3) Entropy	0.762 (0.606–0.919)	2.09	88.9	61.1
Horzl_RLNonUni	0.775 (0.623–0.926)	163.28	55.6	94.4
S (5,0) Entropy	0.790 (0.640–0.940)	2.16	66.7	88.9
S (5,5) Entropy	0.762 (0.608–0.917)	2.04	77.8	61.1
135dgr_RLNonUni	0.772 (0.618–0.926)	191.91	55.6	94.4
S (3,3) AngScMom	0.762 (0.606–0.919)	0.0103	61.1	88.9
S (5,0) SumAverg	0.756 (0.592–0.920)	58.99	94.4	55.6
Superior internal jugular LNs				
WavEnHH_s-3	0.682 (0.506–0.858)	55.09	88.9	50.0
WavEnLL_s-1	0.682 (0.504–0.860)	24,129.76	61.1	77.8
S (1,−1) SumAverg	0.673 (0.494–0.851)	83.61	61.1	77.8
S (1,1) SumAverg	0.682 (0.505–0.859)	83.24	61.1	77.8
S (0,1) SumAverg	0.682 (0.505–0.859)	82.40	61.1	77.8
Perc.99%	0.681 (0.503–0.858)	209.50	61.1	77.8
Perc.90%	0.691 (0.517–0.866)	192.50	61.1	77.8
Perc.01%	0.728 (0.561–0.896)	95.50	61.1	83.3
Kurtosis	0.710 (0.540–0.879)	−0.46	44.4	94.4
Mean	0.679 (0.501–0.857)	161.13	61.1	77.8

Abbreviations: AUC, area under the receiver operating characteristic curve; CI, confidence interval; MRONJ, medication-related osteonecrosis of the jaw; LN, lymph node; AngScMom: angular second moment; SumAverg: sum average; RLNonUni: run-length nonuniformity; Perc.: percentile; WavEnHH: wavelet energy HH.

## Data Availability

The data presented in this study are not publicly available due to privacy and ethical restrictions. Further information regarding the data may be available from the corresponding author upon reasonable request, subject to institutional and ethical approval.

## Abbreviations

MRONJ	Medication-related osteonecrosis of the jaw
MRI	Magnetic resonance imaging
GLCM	Gray-level co-occurrence matrix
STIR	Short tau inversion recovery
TR	Repetition time
TE	Echo time
TI	Inversion time
NEX	Number of excitations
ETL	Echo train length
ROIs	Regions of interest
ROC	Receiver operating characteristic
AUC	Area under the curve
CT	Computed tomography
